# Genetic Stasis of Dominant West Nile Virus Genotype, Houston, Texas

**DOI:** 10.3201/eid1304.061473

**Published:** 2007-04

**Authors:** C. Todd Davis, Li Li, Fiona J. May, Rudy Bueno, James A. Dennett, Adil A. Bala, Hilda Guzman, Darwin Elizondo-Quiroga, Robert B. Tesh, Alan D. Barrett

**Affiliations:** * University of Texas Medical Branch, Galveston, Texas, USA; † Harris County Public Health and Environmental Services, Houston, Texas, USA; ‡ Universidad Autonoma de Nuevo Leon, San Nicolas de Los Garza, Nuevo Leon, Mexico; 1 Current affiliation: Centers for Disease Control and Prevention, Atlanta, Georgia, USA

**Keywords:** West Nile virus, Flavivirus, molecular epidemiology, viral evolution, phylogenetics, dispatch

## Abstract

The accumulation and fixation of mutations in West Nile virus (WNV) led to the emergence of a dominant genotype throughout North America. Subsequent analysis of 44 isolates, including 19 new sequences, from Houston, Texas, suggests that WNV has reached relative genetic stasis at the local level in recent years.

Previous phylogenetic analyses of North American West Nile virus (WNV) isolates have shown genetically distinct variants that group in a temporally and geographically dependent manner (*1*). Recent studies have provided substantial evidence that a dominant genetic variant has emerged across much, if not all, of North America (*2*–*4*). The establishment of a dominant genotype across the continent and the displacement and possible extinction of earlier progenitor genotypes appear to have resulted from the accumulation and fixation of multiple nucleotide mutations throughout the WNV genome. Despite the occurrence of 13 conserved nucleotide mutations (out of 11,029 nt/genome) in isolates belonging to the dominant genotype, only 1 of these mutations, E-1442(U to C); E159(Val to Ala), resulted in an amino acid substitution (out of 3,433 amino acids/polyprotein). Consequently, a scarcity of nonsynonymous mutations in the dominant genotype compared with progenitor genotypes has made it difficult to evaluate and quantify any relative fitness advantages possessed by the dominant strains (*5*,*6*).

The rapid emergence of the dominant genotype across North America and subsequent displacement of other genotypes suggested an apparent fitness advantage conferred by mutations in the genome of the dominant variants. In fact, studies by Ebel et al. suggest that the dominant genotype is transmitted by *Culex pipiens* after fewer days of extrinsic incubation than are needed by the prototypical strain, WN-NY99, leading to a possible increase in the transmission efficiency of the dominant genotype (*6*). The proliferation of this genotype over such a relatively short period (≈3 years, from the summer of 1999 through the summer of 2002) and vast geographic scale has led us to consider whether the genetic divergence of the virus has continued at a similar rate. To further characterize the evolutionary patterns of WNV, we chose to focus on a readily available population of virus isolates that provide a representation of the current state of WNV evolution at a localized level.

## The Study

The 44 WNV isolates included in this study were obtained in the Houston, Texas, metropolitan area during the course of 5 years (2002–2006). All of the 25 earlier isolates and 19 newly sequenced isolates came from dead birds or mosquitoes and were isolated as previously described (*7*). The complete premembrane (prM) and envelope (E) protein genes (2004 nucleotides) were sequenced by reverse transcription–PCR (RT-PCR) with RNA extracted from cell culture supernatant of either the original isolation or after a single passage in Vero cells. RT-PCR protocols, primer sequences, and sequencing methods have been described elsewhere and are available on request (*1*). Nucleotide and deduced amino acid sequences of all isolates were aligned with the prototypical North American WNV, WN-NY99, by using AlignX in the VectorNTI software package (Informax, Frederick, MD, USA). The year, source, and corresponding GenBank accession number for each isolate are described in Table 1. A phylogenetic tree was generated by maximum likelihood analysis by using PAUP (*8*) (Figure).

**Table 1:** West Nile virus isolates, Houston, Texas, 2002–2006

Isolate	Year of isolation	Source	GenBank accession no.
Bird 113	2002	Bluejay	AY185906
Bird 114	2002	Bluejay	AY185907
Bird 119	2002	Bluejay	AY185908
Bird 123	2002	Hawk	AY185909
Bird 135	2002	American crow	AY185910
v1151	2002	*Culex quinquefasciatus*	AY185911
Bird 227	2002	Bluejay	AY185912
Bird 1519	2003	Bluejay	DQ158227
Bird 1574	2003	Bluejay	DQ158228
Bird1576	2003	Bluejay	DQ158229
Bird 1461	2003	Bluejay	AY712947
Bird 1153	2003	Mourning dove	AY712945
Bird 1171	2003	Great-tailed grackle	AY712946
Bird 1175	2003	Bluejay	DQ158220
Bird 1240	2003	Bluejay	DQ158221
Bird 9045	2003	Bluejay	DQ158223
Bird 9114	2003	Bluejay	DQ158222
v4095	2003	*C. quinquefasciatus*	DQ158224
v4369	2003	*C. quinquefasciatus*	AY712948
v4096	2003	*C. quinquefasciatus*	DQ158226
v4370	2003	C. quinquefasciatus	DQ158225
Bird 2541	2004	*C. quinquefasciatus*	DQ158234
Bird 2419	2004	Bluejay	DQ158233
Bird 3588	2004	Bluejay	DQ164206
Bird 3218	2004	Bluejay	DQ158235
M8447†	2004	*C. restuans*	EF205419
M8451†	2004	*C. restuans*	EF205420
M8977†	2004	*C. restuans*	EF205421
Bird 4511†	2005	American crow	EF205422
Bird 4486†	2005	American crow	EF205423
Bird 4276†	2005	Bluejay	EF205424
Bird 4487†	2005	American crow	EF205425
Bird 5001†	2005	Bluejay	EF205426
Bird 5014†	2005	Bluejay	EF205427
Bird 5055†	2005	House sparrow	EF205428
Bird 5058†	2005	Bluejay	EF205429
M11769†	2005	*C. quinquefasciatus*	EF205430
M12214†	2005	*C. quinquefasciatus*	EF205431
M12251†	2005	*C. quinquefasciatus*	EF205432
M12357†	2005	*C. quinquefasciatus*	EF205433
M1977†	2006	*C. quinquefasciatus*	EF205434
Bird 5784†	2006	Bluejay	EF205435
Bird 5810†	2006	Common grackle	EF205436
M2766†	2006	*C. quinquefasciatus*	EF205437

*Isolates IS98STD1 (GenBank accession no. AF481864) and WN-NY99 (GenBank accession no. AF196835) were used during phylogenetic analysis. †Isolate was sequenced for this study.

**Figure:**
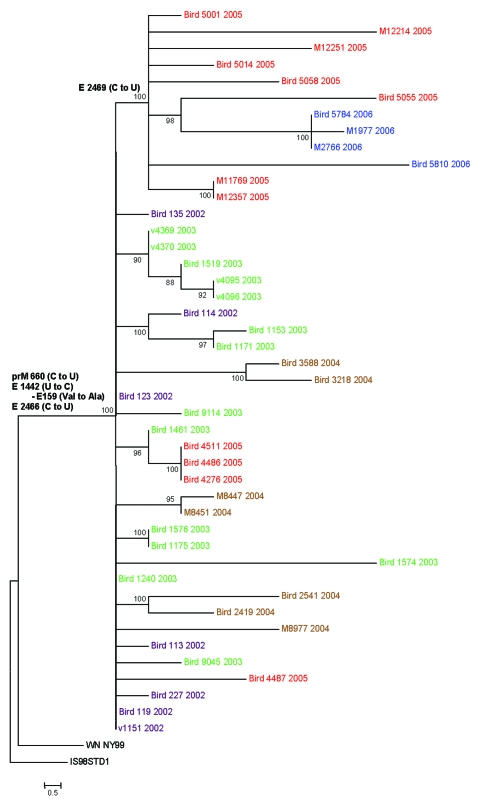
Phylogenetic tree generated by maximum likelihood analysis of a nucleotide alignment of the premembrane and E protein genes (2004 nucleotides) of previous and newly sequenced West Nile virus (WNV) isolates collected in the Houston metropolitan area from 2002 to 2006. The tree was rooted with the most closely related Old World WNV strain, Israel-1998. Maximum likelihood analysis was used to generate trees using PAUP (Version 4.0b11, Sinauer Associates, Sunderland, MA, USA) under the general time-reversible model and a γ distribution of substitution rates with statistical support and tree topology confirmation provided by 1,000 bootstrap replicates (bootstrap values shown at each node). Parsimony informative nucleotide mutations and deduced amino acid substitutions responsible for the observed clade topologies were added to the tree at relevant nodes. The year of isolation is color coded for each isolate on the tree (2002, purple; 2003, green; 2004, brown; 2005, red; 2006, blue), and the scale bar represents 0.5 nt changes.

Not surprisingly, all of the isolates analyzed in this study contained the 3 nt mutations in the prM and E protein genes that differentiate the dominant genotype from other genotypes (prM-660[C to U]; E-1442[U to C]; E-2466[C to U]), which places each of the newly sequenced isolates in the dominant genotype clade relative to WN-NY99. Although several isolates shared additional mutations, most made up a large, indistinct polytomy with little branching and subclade formation. Most isolates from 2002 had the shortest branch lengths from the node separating the dominant clade from WN-NY99, which supports the hypothesis that the 2002 isolates represented the early stages of the emergence of the dominant genotype. The isolates from 2003, 2004, and 2005 had, on average, longer branch lengths than those of 2002, but few had >3 nt mutations from the dominant clade defining node, which suggests relative stasis in the divergence of the virus.

Of particular note was the presence of a subclade at the apical portion of the phylogenetic tree, composed of only isolates from 2005 and 2006. This distinct subclade was the result of a conserved silent nucleotide mutation at position E-2469(C to U), which had not been identified in any other publicly available WNV sequences (data not shown). The four 2005 isolates that were not included in this subclade (i.e., that do not have this mutation) were collected in the spring (March/April), while all other 2005 isolates were collected in early summer (June/July), suggesting that this mutation occurred during the 2005 WNV season but did not become fixed in the population until at least the beginning of the annual peak transmission period (June–September). Alternatively, this mutation may have already been present in a virus introduced into the Houston area during early summer 2005. Regardless, the fixation of this mutation in isolates from 2005 and 2006 indicated its presence in the population at the start of intense WNV transmission in 2005, and it may have become fixed not as a result of positive selection or an increase in fitness but simply because it was present in those viruses that were subjected to an increased frequency of transmission during the early summer months.

To demonstrate the degree to which isolates have diverged from year to year, nucleotide sequences were grouped by year and the average pairwise distances between groups were calculated. Table 2 shows that the percent nucleotide divergence from WN-NY99 has generally increased over time (2002, 0.29%; 2006, 0.60%). Surprisingly, isolates from 2005 were on average slightly less divergent from WN-NY99 (0.45%) than were 2004 isolates (0.46%). Analysis of nucleotide sequence alignments showed that the 2005 isolates did not share any mutations with the 2004 isolates other than those denoting the dominant genotype (data not shown), which suggests that the 2005 isolates did not acquire mutations from viruses circulating during the previous transmission season. In addition, as shown in Table 2, the nucleotide divergence has marginally increased from year to year (2002–2003, 0.15%; 2003–2004, 0.32%; 2004–2005, 0.41%; 2005–2006, 0.48%). This finding indicates that, while WNV has continued to diverge, most mutations that occur each year are not passed on, and even viruses circulating in the same location continually diverge from 1 another if mutations are not fixed from year to year.

**Table 2:** Average pairwise percent nucleotide divergence between groups by year

Group	ISR 1998	WN NY99	2002	2003	2004	2005	2006
ISR 1998							
WN NY99	0.20						
2002	0.29	0.29					
2003	0.36	0.36	0.15				
2004	0.46	0.46	0.24	0.32			
2005	0.45	0.45	0.24	0.31	0.41		
2006	0.60	0.60	0.38	0.46	0.56	0.48	

## Conclusions

The relative stasis of the Houston metropolitan area WNV population and lack of newly emergent subclades containing 2002, 2003, and 2004 isolates suggest that few, if any, new genotypes of WNV from other regions of North America have been introduced locally and that the dominant genotype has been established and maintained in a local endemic transmission cycle. Additional sequence data from outside the Houston area collected during the same sampling period will be necessary to confirm or refute this hypothesis.

Of the 16 deduced amino acid substitutions that have occurred in the population studied (data not shown), only a single substitution (E159-Val to Ala) has become fixed, an indication that the dominant variant may be in a period of relative stasis. Only a single, silent nucleotide mutation has become fixed in the population since 2002, which indicates the infrequency of such molecular events and may reflect restrictions imposed on the genome; alternatively, it may indicate a lack of positive selective pressures acting on the virus population. During such intervals, if no fitness advantage is conferred by a particular substitution, random mutations may continue to accumulate at low frequencies, eventually giving rise to new subclades as a result of fixation. In this case, fixation may be due to the increased rate of transmission of a particular virus population during intensified transmission periods (i.e., after an increase in mosquito density), rather than in response to selective pressures or increased viral fitness. Thus, the forces driving the evolution of WNV may differ from location to location, and the newly described stasis of the Houston WNV population described in this study may or may not reflect similar trends in the continuing evolution of the virus in other regions of North America. Additionally, it is notable that there has been a lack of genetic divergence in an area lying on a major migratory bird flyway. The epidemiologic consequences of stasis within the Houston WNV population may have important implications for the endemicity of WNV disease in the Houston area and other similar locations.
